# Studies of the macroscopic and microscopic morphology (hippocampus) of brain in Vencobb broiler

**DOI:** 10.14202/vetworld.2016.507-511

**Published:** 2016-05-24

**Authors:** Shailesh Kumar Gupta, Kumaresh Behera, C. R. Pradhan, Arun Kumar Mandal, Kamdev Sethy, Dayanidhi Behera, Kuladip Prakash Shinde

**Affiliations:** 1 Department of Livestock Production and Management, College of Veterinary Sciences and Animal Husbandry, Bhubaneswar - 751 003, Odisha, India; 2 Department of Veterinary Anatomy and Histology, College of Veterinary Sciences and Animal Husbandry, Bhubaneswar - 751 003, Odisha, India; 3 Department of Animal Nutrition, College of Veterinary Sciences and Animal Husbandry, Bhubaneswar - 751 003, Odisha, India; 4 Livestock Production Management Section, ICAR - National Dairy Research Institute (NDRI), Karnal - 132 001, Haryana, India

**Keywords:** capillaries, hemisphere, hippocampus, neurons, Vencobb broiler

## Abstract

**Aim:**

The aim of this study was to study the anatomy of different parts of brain and histology of hippocampus of Vencobb broiler chicken.

**Materials and Methods:**

A 12 adult experimental birds were sacrificed by cervical dislocation. After separation of the brain, gross anatomy features were studied. Brain tissue was fixed in 10% buffered neutral formalin for 2-3 days, and then routine dehydration process in ascending grades of ethyl alcohol was done. After xylene cleaning, paraffin impregnation was prepared. Paraffin blocks were cut, and slides were stained by Harris hematoxylin and eosin. Photography was carried out both under lower (×10) and higher (×40) magnifications.

**Results:**

The brain structure (dorsal view) of Vencobb bird resembled the outline of a playing card symbol of a “spade.” The brain subdivisions are cerebrum, cerebellum, and medulla oblongata. Cerebrum was devoid of usual convolutions (elevations), gyri, depressions (grooves), and sulci. The cerebral hemispheres were tightly apposed along a median sulcus called interhemispheric fissure and cerebrum and cerebellum were separated by a small transverse fissure. The olfactory bulb was small structures, and the pineal body was clearly visible. The optic lobes were partially hidden under cerebral hemispheres, but laterally, it was large, prominent rounded or spherical bodies of the midbrain. The hippocampal area appeared as dorso-medial protrusion. Different types of neurons were distinguished in the hippocampus were pyramidal neurons, pyramidal-like neurons, and multipolar neurons, etc. There was rich vascularization in the form of blood capillaries throughout the hippocampus.

**Conclusion:**

Cerebrum was pear shaped and largest part of the brain. Cerebrum hemisphere was smooth devoid of convolutions, gyri, and depressions, but in the surface of cerebellum, there was the presence of a number of transverse depression (grooves) and sulci subdividing into many folds. Olfactory bulb was poorly developed, whereas optic lobes were rounded and large. The exact boundary line of the hippocampus was not discernable. In hippocampus histology, two categories of neuron local circuit neurons and projection neurons, high vascularization and epididymal lining of lateral ventricle were observed. Hippocampal neurons were comparatively larger without any distinct layers. The afferent neurons projected to the medium septum.

## Introduction

Avian brain research was started in the early 20^th^ century. Brain research in birds is important for bird’s welfare and knowledge about the nervous system function, physiology, anatomical development, and behavior. The cognitive ability of a species might be due its total number of brain neurons [1]. Large brains might have evolved as an adaptation to cope with novel or altered conditions. The large optic lobes in avian species can be attributed to the fact that the birds have a very well-developed sense of vision [2]. There is wide variation in brain morphology among different birds. The brain was hour-glass shape and large in the white crested polish chicken and cerebellum, hippocampus, septum, and olfactory bulb were well developed [3]. Smaller birds tend to have round and avian-type brains, whereas larger birds show elongated and reptilian-type brains [4]. Sturnus vulgaris birds showed that the cerebral is an oval shape with the absence of gyrus and sulcus, with right and left cerebral hemisphere separated by medium fissure, whereas another transverse fissure situated between cerebral and cerebellum [5]. The cerebral cortex of the domestic fowls is similar to that of the Pekin Duck and African ostrich [6].

The role of the avian hippocampus in spatial learning, memory, cognitive, and navigation is well established. Five fields were recognized in the hippocampal complex: Medial and lateral hippocampus, parahippocampal area, central field of the parahippocampal area, and crescent field [7]. Several types of local circuit neurons (LC) beside the three types of projection neurons: Pyramidal, pyramidal-like, and multipolar neuron have been described in domestic chicks [8]. In case of strawberry finch, *Estrada amandava* birds several types of neurons present in the hippocampal complex on the basis of differential dendritic tree pattern [7].

Till today little work has been published regarding anatomical and histological study of brain in birds. So the present study was designed to investigate the macroscopic and microscopic morphology (hippocampus) of brain in Vencobb broiler. This work will help to find more information related to brain in Vencobb broiler.

## Materials and Methods

### Ethical approval

The present retrospective study was duly approved by the Institutional Animal Ethics Committee (IAEC), OUAT, College of Veterinary Science and Animal Husbandry, Bhubaneswar, Odisha-751003.

### Sample collection and staining

About 12, day old Vencobb chicks were obtained from Eastern Hatchery, Bhubaneswar, Odisha, India. Birds were reared up to 8 weeks with standard housing, feeding, vaccination, and management system. Adult birds (8 weeks) were slaughtered by cervical dislocation. The head of the birds under study was carefully separated at the level of second cervical vertebrae. Immediately, the separated head of these birds was taken to the laboratory of the Department of Veterinary Anatomy and Histology. The cranial cavity was cut open very carefully with the help of forceps, scissors, and scalpel. The meanings covering of the brain and its attachment with cranial bones was cut followed by serving of anterior/rostral attachment of olfactory lobes and optic nerves at the level of optic chiasma on the ventral surface of the brain; the intact brain was removed from the cranial cavity. After the collection, the brain samples were cleaned (washed) in normal saline solution then the gross anatomical study was done. Small tissue pieces were collected from dorsomedial (DM) part of each cerebral hemisphere through the transverse section; the representative tissue pieces were immediately fixed in 10% buffered neutral formalin for 2-3 days before tissue processing. The tissue pieces were washed under slow running tap water for an overnight period, followed by routine dehydration process in ascending grades of ethyl alcohol (70% → 80% → 90% → absolute alcohol) for 45 min to one hour in each change. Thereafter, the tissue was cleared in two changes of xylene (4-5 h in each) followed by paraffin impregnation in a thermostatically controlled oven to prepare the paraffin blocks. The trimmed paraffin blocks were cut with the help of a semi-motorized rotary microtome (Leica RM 2245™) to obtain 5-7 μm thick serial paraffin section. The tissue sections were mounted on clean, grease free, albumenized glass slides. After air drying, the slides were kept on a slide drier for better fixation of the section. Finally, the desired slides with tissue section were stained by Harris hematoxylin and eosin as per the standard method [9]. Photography was carried out from the selected fields both under lower (×10) and higher (×40) magnifications under (Leica DM 2500, Germany) microscope.

## Results and Discussion

### Gross anatomical study of brain

**Figure-1:**
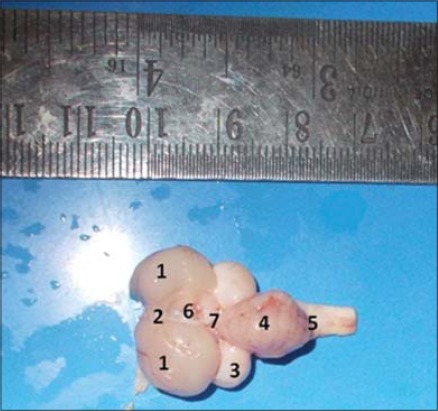
Dorsal view of brain showing different gross anatomical parts (1) Cerebral Hemisphere, (2) Interhemispheric Fissure, (3) Optic Lobe, (4) Cerebellum, (5) Medulla Oblongata, (6) Pineal body, (7) Transverse Fissure.

**Figure-2:**
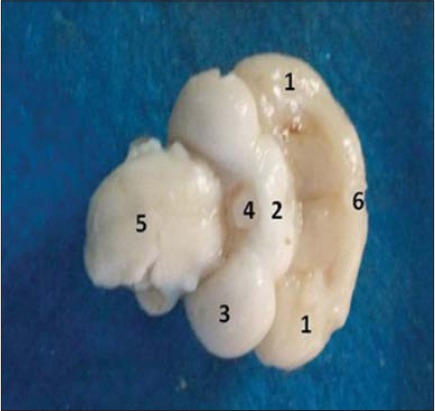
Ventral view of brain showing different gross anatomical parts. (1) Cerebral hemisphere, (2) Optic chiasma, (3) Optic lobe, (4) Hypothalamus, (5) Medulla oblongata, (6) Olfactory bulb.

After careful removal of meninges covering, the gross anatomical features of the brain were observed. The general appearance of the brain (dorsally) resembled the outline of a playing card symbol of a “spade.” The finding on the shape of brain corroborates well with the observations in barn owl [2], in locally bred chicken [10], and in migratory bird [5]. They mentioned that the general shape of the avian brain was more or less triangular or pear shaped. The brain consisted of three major subdivisions: Cerebrum, cerebellum, and medulla oblongata. The cerebrum was pear shaped or obtuse triangle like in Vencobb birds (Figure-1). Cerebrum was well developed and the largest part of the brain in experimental birds. These observations are in accordance with the reports in both white crested polish chickens and uncrested chicken breeds [3]. Cerebrum comprised two symmetrical cerebral hemispheres (right and left) (Figures-1 and 2). The cerebral hemisphere shape is an important determining factor for the shape of the entire brain. Even it may vary with shape and size of the large eye and orbit [4]. The dorsal surface of the cerebral hemispheres was moderately convex and more or less smooth contoured as it was devoid of usual convolutions (elevations), gyri, depressions (grooves), and sulci. The finding corroborates well with finding in Sturnus vulgaris birds [5]. Caudal part of each hemisphere gradually became much wider than its narrow rostral (anterior) tip. The cerebral hemispheres were tightly apposed along a median sulcus called interhemispheric fissure. The cerebrum and cerebellum were separated by a small transverse fissure similar as the previous finding (Figure-1) [5]. The olfactory bulbs were relatively small structures of poorly developed rhinencephalon at the rostral pole of the hemispheres. Because of its intimate connection with olfactory nerve (neurons), the olfactory bulbs in many specimens appeared distorted during collection. On either side of the interhemispheric (longitudinal) fissure was a slight enlargement called sagittal eminence (Wulst), (Figure-1) whose lateral curved margin was demarcated from the rest of the hemispheric surface by an indistinct groove (vallecula). The sagittal eminence became flattened and continued to the caudal (posterior) pole of the hemisphere. However, the presence of indistinct groove (vallecula) and relatively small sagittal eminence was in contrary in ostrich [6] and barn owl [2], who observed very large wulst and distinct vallecula in the brain. The entire optic lobes were not visible on dorsal view. The optic lobes were partially hidden under cerebral hemispheres (Figure-1). However, these optic lobes, i.e. tectum were very large, prominent rounded or spherical bodies of the midbrain on the lateral view. The finding was corroborated well with the previous observations [11]. A small pineal body was clearly visible at the posterior end of the interhemispheric fissure (Figure-1). The cerebellum was large, laterally compressed, wedge-shaped structure posterior to cerebrum that formed the major part of the hindbrain (rhombencephalon). Both the anterior and posterior ends were comparatively narrower than its middle part (Figure-1). The presence of large cerebellum was in contrary to the observation in swifts and falcons birds as they have small cerebellum [12]. The cerebellum extended a backward covering the most part of the medulla oblongata. The surface of the cerebellum presented a number of transverse grooves (sulci) subdividing it into many folds (folia).

**Figure-3:**
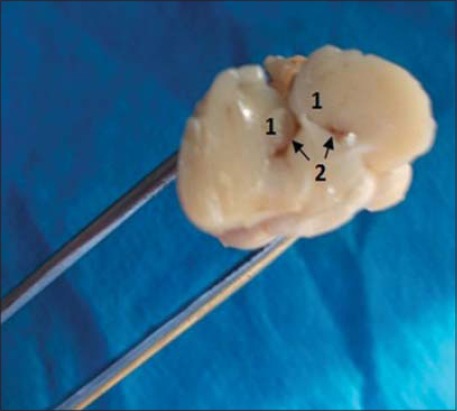
Transverse section of brain. (1) Hippocampus, (2) Lateral Ventricle.

On ventral view, the poorly developed olfactory lobes (rhinencephalon) were observed. The finding was corroborated well with the previous observations [11] and contrary to finding in white crested polish chicken [3]. The optic chiasma (X-shaped point of criss-crossing/exchange of optic nerve fibers), which was present just posterior diencephalon (hypothalamus and its connection with pituitary gland/hypophysis) (Figure-2). However, none of the brain specimens showed intact hypophysis and its attachment. A transverse section was made through the posterior 1/3^rd^ part of both the cerebral hemispheres to locate the hippocampus in respect to the respective lateral ventricle. The hippocampal area appeared as DM protrusion/elevation into the narrow, slit-like lateral ventricle on either side (Figure-3). However, the exact boundary line of the hippocampus was not discernable as earlier has been reports [13].

### Histology of hippocampus

**Figure-4:**
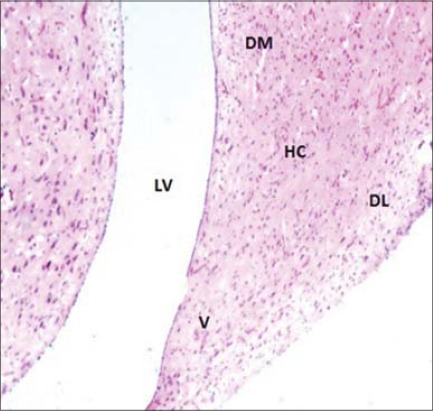
Cross section of hippocampus (HC) & adjacent Lateral Ventricle (LV) (H & E ×100). (DL) Dorso-lateral, (DM) Dorso-medial, (V) Ventral.

Our prime focus of the histological study was hippocampus and adjacent associated structure (lateral ventricle), i.e. hippocampal formation. Hippocampus was separated from the rest of the hemisphere by the presence of a narrow slit-like lateral ventricle (Figure-3). The hippocampal area protruded above the lateral ventricle was subdivided into 3 parts: Dorsolateral, DM, and ventricle (V) (Figure-4). The topographical location (in respect to lateral ventricle) of the hippocampus and its three subdivisions are exactly same as earlier reports in barn owl [2] in laying hens [14], in strawberry finch [7], and in different avian species [13].

**Figure-5:**
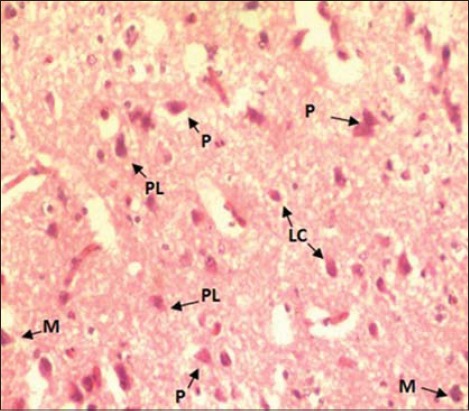
Cross section of hippocampus (H & E ×400) showing different types of neurons. (P) Pyramidal, (M) Multipolar, (PL) Pyramidal like cells, (LC) Local circuit neuron).

**Figure-6:**
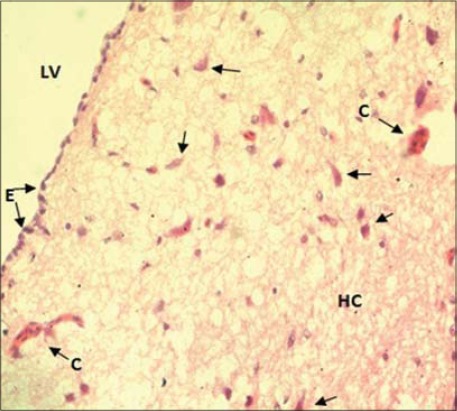
Cross section of hippocampus (HC) (H & E ×400) showing (arrows) Neurons, (C) Capillaries and (E) Ependyma, (LV) Lining of the lateral ventricle.

Histomorphologically, two broad categories of neurons populated the hippocampus: The projection neurons and the LC. Hippocampal neurons in this study did not reveal any distinct cell layer as earlier observed in barn owl [2]. Three visible cell layers in the hippocampus of strawberry finch observed using special Cresyl Violet and Golgi (silver impregnation) staining [7]. They were even able to observe other cell population such as mono and bitufted neurons and radial glial cells in hippocampus. The projection neurons were the predominant cell group included different subtypes: Pyramidal neurons (P), pyramidal-like neurons (PL), and multipolar neurons (M) (Figure-5). Similar neuronal cell types were identified in different birds [2,7,8]. The pyramidal neurons were characterized by their unique pyramidal or triangular shaped medium to the large cell body (soma) with single, thick apical dendrite facing toward piamater (surface). The appearance shape and size of “pyramidal-like cells” were very similar to the pyramidal neurons with poorly developed apical dendrites and the axon facing the lateral ventricle. The multipolar neurons showed 4-5 thick dendrite branches toward different directions after their origin from medium to large sites soma (cell body). Their axons were usually oriented toward the ventricular surface (Figure-5). The LC had small to medium size ovoid perikarya (cell body) and were interspersed with other types of neurons of the hippocampus. The cells were almost like multipolar or even bipolar neurons in appearance (Figure-5). Some of these cells were close/adjacent to pyramidal and multipolar neurons. The axons of the projection neurons joined to form the fiber bundle running parallel to the lateral ventricle. Similar hippocampal projections were studied earlier by several workers in different species of birds [7,8]. These are efferent (outgoing) neuronal projections from the hippocampus to the median septum [7]. Previously, it was reported that hippocampal efferent projections extend also to the hypothalamus and even directly to cerebellum [15]. Other general histological features of the hippocampus in the current study, i.e. high vascularization in the form of capillaries (C) throughout the hippocampus and ependymal lining of lateral ventricle were also reported previously [2] (Figure-6). In our study, the hippocampal neurons appeared comparatively larger (cell body size) under high magnification.

## Conclusion

In conclusion, cerebrum was pear shaped and largest part of the brain. Cerebral hemispheres were smooth without convolutions (elevations), gyri, depressions (grooves), and sulci, but in the surface of cerebellum, there was the presence of a number of transverse depressions (grooves) and sulci subdividing into many folds. Olfactory bulb was poorly developed, whereas optic lobes were rounded and large in size. Hippocampus appeared as DM protrusion, but the exact boundary line of the hippocampus was not discernable as previous reports. In histological studies, two categories of neuron LC and projection neurons (P, PL, and M), high vascularization and epididymal lining of lateral ventricle were observed. Hippocampal neurons are comparatively larger in size without any distinct layers. The afferent neurons projected to the medium septum. Further study may be directed to the use of more advanced techniques for tissue section preparation and staining of the brain tissue.

## Authors’ Contributions

SKG, KB, and CRP designed the plan of work. AKM, SKG, and KB performed laboratory investigation. KS and DB helped in the laboratory investigations. SKG, KB, AKM, and KPS participated in draft and revision of the manuscript. All authors read and approved the final manuscript.
